# A Multilevel Study of Students in Vietnam: Drinking Motives and Drinking Context as Predictors of Alcohol Consumption

**DOI:** 10.3390/ijerph13070710

**Published:** 2016-07-13

**Authors:** Pham Bich Diep, Frans E. S. Tan, Ronald A. Knibbe, Nanne De Vries

**Affiliations:** 1 Department of Health Promotion, CAPHRI School for Public Health and Primary Care, Maastricht University, P.O. Box 616, Maastricht 6200 MD, The Netherlands; r.knibbe@maastrichtuniversity.nl (R.A.K.); n.devries@maastrichtuniversity.nl (N.D.V.); 2 Department of Methodology and Statistics, CAPHRI School for Public Health and Primary Care, Maastricht University, Debeyeplein 1 (1st Floor), Postbus 616, Maastricht 6200 MD, The Netherlands; frans.tan@maastrichtuniversity.nl

**Keywords:** drinking motives, drinking context, alcohol intake, students, Vietnam

## Abstract

*Background*: This study used multi-level analysis to estimate which type of factor explains most of the variance in alcohol consumption of Vietnamese students. *Methods*: Data were collected among 6011 students attending 12 universities/faculties in four provinces in Vietnam. The three most recent drinking occasions were investigated per student, resulting in 12,795 drinking occasions among 4265 drinkers. Students reported on 10 aspects of the drinking context per drinking occasion. A multi-level mixed-effects linear regression model was constructed in which aspects of drinking context composed the first level; the age of students and four drinking motives comprised the second level. The dependent variable was the number of drinks. *Results*: Of the aspects of context, drinking duration had the strongest association with alcohol consumption while, at the individual level, coping motive had the strongest association. The drinking context characteristics explained more variance than the individual characteristics in alcohol intake per occasion. *Conclusions*: These findings suggest that, among students in Vietnam, the drinking context explains a larger proportion of the variance in alcohol consumption than the drinking motives. Therefore, measures that reduce the availability of alcohol in specific drinking situations are an essential part of an effective prevention policy.

## 1. Introduction

Heavy drinking among students in developed countries is well documented. In 2011, a study including 36 European countries showed a prevalence of drinking five or more drinks on one occasion in the last 30 days of 27%–61% among male students and 12%–53% among female students [1]. However, very few studies have investigated young people in developing countries [2] and, in particular, in Vietnam [3]. Available studies among medical students in Vietnam showed that 15.1% of students were abstainers; 65.5% of students consumed alcohol in the past year of which 12.5% reported alcohol-related problems; 6.1% of the female students reported drinking at least four standard drinks per occasion and 36.4% of male students drank at least five standard drinks per occasion; and male students were 14.3 times more likely to have alcohol-related problems than female students [4,5]. Factors associated with alcohol consumption in Vietnam have not yet been described. Therefore, this study examines the effects of individual factors (age and drinking motives) and contextual factors (drinking with whom, drinking duration, drinking relationship, drinking location, main purpose of drinking, drinking with food) on alcohol intake per occasion among university students in Vietnam.

Conceptually, environmental factors are important for etiological explanations of drinking behavior [6]. Individuals might not drink in the same way on different occasions, and actual drinking might be as much the result of the individual adjusting his/her drinking to situational aspects as to individual characteristics explaining his/her drinking in a particular situation [7]. Studies using self-reported data found that some aspects of the drinking context are associated with alcohol consumption among students: aspects of timing (weekend; duration of drinking sessions) [8]; social aspects (gender composition, size of group, relation with drinking group members, number of intoxicated people) [9,10,11]; and aspects of location (bar or tavern; off-campus party) [12,13,14]. Additionally, observational studies of drinking situations also show similar results in terms of social aspects, such as being in a larger group, in a group of mixed/male gender; loudness of the music [15,16]; poor cleanliness, loud music and crowdedness [17].

Drinking context is very rarely studied in Vietnam. Only one study among young people aged over 15 years in three provinces in Vietnam showed that most people drink in the late afternoon (87% for wines/spirits and 31% for beer). In terms of the drinking place, the most frequently reported location was at home (55.5% when drinking wine and 22.2% when drinking beer), followed by parties/weddings (39.6% for wine and 16.4% for beer) and street bars (11.6% when drinking wine and 12.5% for beer) [18].

While the study of contextual aspects is based on the assumption that the individual adjusts their drinking to the situation, most theories (such as motivational or expectancy theory) assume that individual characteristics have the strongest influence on drinking. The assumption of this model is that motives are the most proximate determinant of an individual’s drinking and that other factors, like biochemical factors, personality characteristics, situational and other cognitive factors should be considered as antecedent factors, which are reflected in the motives of drinking [19,20,21]. It has been suggested that motives mediate the influence of other important individual factors like expectancies [22,23,24,25]. For young people, the most dominant type of motive is social drinking motive, followed by enhancement, coping and conformity motives [26]. The different motives are associated with different aspects of drinking patterns. Social motives are strongly and positively related to drinking frequency [26]; enhancement and coping motives are positively associated with heavy drinking and alcohol-related problems [27,28], while conformity motives are negatively associated with alcohol use [26].

The present study is based on the assumption that both drinking motives and drinking context characteristics are explanatory variables for alcohol consumption. Individuals might seek to participate in drinking environments that are consistent with their motivations. On the other hand, individuals interact with each other and the drinking environment that they are participating in. Therefore, the same individual may drink differently on different occasions [11,16]. Drinking motives and drinking context may influence each other in two ways: motives may determine the selection of the drinking contexts someone drinks in and, depending on the drinking context, a particular motive may be more prominent in determining drinking behavior than it is in another context. However, this relationship has not yet been studied. As a first step, this study determines how much variance in alcohol intake per occasion is explained by drinking context characteristics and by individual characteristics, such as motives.

A few multilevel studies included context and individual levels in order to examine the association with alcohol consumption. Kypri et al. [14] studied alcohol use as a function of only one situational variable (i.e., drinking location) and of individual characteristics (age, living arrangement, drinking history, etc.). College students drinking before, during and after they went to a certain setting, were also studied in relation to the socio-demographic characteristics [29]. The results show a positive association between drinking settings and alcohol use. A multilevel analysis among American students combined observational measures of party environment (party size, loud music, exit time, number of intoxicated people, etc.) with a brief self-administered questionnaire about individual factors (gender, motivation to go to the party, etc.) and collected breath samples from partygoers. It was shown that party characteristics explained more variance in blood alcohol concentration at both the individual and party level, than the individual variables [13]. This study examines to what extent drinking motives, indicating individual characteristics, and drinking context characteristics explain alcohol consumption per occasion. 

## 2. Method

### 2.1. Setting

This study involved 6011 students from the first to the last academic years of total 10 universities from four provinces: Hanoi (HN) in the north; HoChiMinh (HCM) in the south; Hue in the middle and BuonMeThuat (BMT) in a remote region of Vietnam. In three of the cities (HN, HCM, Hue) the three largest universities focusing on Economics, Technology and Medicine were selected for this study. BMT has only one university; therefore, in that university the three faculties focusing on Economics, Technology and Medicine were selected. 

### 2.2. Sampling

A multi-stage sampling strategy was used with the city as the first stratum, the university/faculty as the second stratum, and the academic year (from the first to the last academic year) the third stratum. Since medical undergraduate education takes six years to complete, while the technological and economics/nature, and the technology science/agriculture & forestry undergraduate education takes only five and four years, respectively, the numbers of students in each academic year in those universities/faculties were 85 for medical, 100 for technological, and 125 for economic studies. Within each academic year, one or two classes (depending on the total number of students per class) were randomly selected for this study, ensuring that the sample size per academic year was sufficient. As a result, 6011 students who were in the classes at the data collection time all agreed to participate in the research. 

### 2.3. Data Collection

The structured questionnaire contained pictures of the most common beverages in Vietnam (with their ethanol levels, and number of standard drinks (SDs) per container (bottle, can, etc.)). In this way students were supported to correctly report the number of drinks. Standard drink (SD) = 1 can beer (330 mL at 5%) = 1 glass wine (140 mL at 12%) = 1 shot spirit (40 mL at 40%) = 12.6 g of pure alcohol. Investigators instructed students on how to fill out the questionnaire. The students were assured of their right to withdraw from the study. If a student had any queries related to the questions, the investigators provided clarification. This study was approved by the Biomedical Research Ethics Committee of School of Public Health in Hanoi, Vietnam (Code: 136/2012/YTCC-HD3). 

### 2.4. Questionnaire

*General information* concerned the city; the university/faculty; gender and age.

*Outcome variable*: Number of glasses reported for each of the three drinking occasions is the outcome variable in our analysis. Since students will considerably differ in the time span over which these three occasions were distributed, this measure is not indicative of level of consumption understood in the more common way as for example number of glasses per week or month. It appears that the correlation of number of glasses per occasion with consequences due to drinking (measured with the Alcohol Use Disorders Identification Test (AUDIT) questionnaire) is about the same as the correlation of the correlation of level of consumption (defined by AUDIT questions on frequency of drinking and number of glasses per drinking day and consequences. For men these correlations were 0.43 (number of glasses per occasion) and 0.49 (AUDIT measure of volume), respectively and the correlations for women were 0.36 and 0.41, respectively. Therefore the informative value of our outcome variable for experiencing negative consequences due to drinking is about as good as an outcome defined in terms of level of consumption.

*Drinking behavior and context*: Each student was asked: “How often did you drink in the last 12 months” with the five answers of categories: never/maximum of 1 time a month/2–4 times a month/2–3 times a week, and at least 4 times a week. Students who answered “never” in the last year (*n* = 1021) and who answered “no” to the question “Did you drink at least three times in the last year” (*n* = 725) were excluded from the analysis. As a result, 4265 individuals who drank on at least three occasions remained in the analysis.

*Drinking context characteristics*: Students were asked to name and describe the last three occasions at which they drank; there were 12,795 drinking occasions in the analysis. For each drinking occasion, students described how many SDs they consumed; the drinking day (week days, weekend, holiday); the time of day (morning/lunch, afternoon, evening/midnight), duration of drinking (≤1 h, 1–2 h, 2–3 h, ≥3 h); main purpose of drinking occasion (party, meeting of friends, specific events (e.g., wedding or other celebration), and no specific purpose); drinking with food, even a snack (yes, no); drinking location (at home, in a restaurant, tavern/at the pavement/outside, in a discotheque/karaoke bar/others); number of people that they drank with (none, 1–3 people, 4–9 people, more than 10 people); the gender of the people they drank with (male only, female only, mixed gender); and the relationship with the people they drank with (family members/relatives, friends, casual relationship).

*Drinking motives:* For this study we used the Drinking Motive Questionnaire Revised, as developed by Cooper [30].This was found to be a valid and reliable instrument in different age groups and has also been used in non-English speaking countries. The questionnaire on drinking motives was first translated from English into Vietnamese (by the first author) and then back translated into English by professional native-speaking persons. Also, before conducting the study, the questionnaire was piloted among students to ensure that they correctly understood the included questions. Students were asked to indicate on a 5-point Likert scale ranging from “Never = 1” to “Almost always = 5” how often they consumed alcohol for each of the 20 motive items. Exploratory factor analysis detected four factors covering five items per factor, similar to the dimensions of Cooper, all with adequate internal consistency: Social (Cronbach’s alpha = 0.80), Enhancement (Cronbach’s alpha = 0.87), Coping (Cronbach’s alpha = 0.85) and Conformity (Cronbach’s alpha = 0.71). The average score of the relevant items was used as the scale score. Male students scored significantly higher than females on social (Median; interquartile range (IQR) = 3.4 (2.8, 3.8) vs. 3.0 (2.2, 3.6)), enhancement (1.6 (1.2, 2.2) vs. 1.4 (1.0, 1.8)) and coping motives (1.4 (1.0, 2.0) vs. 1.2 (1.0, 1.8)), as well as on conformity motives (2.0 (1.6, 2.6) vs. 1.6 (1.4, 2.0)).

### 2.5. Missing Value Imputation

The missing observations are reported in Table 1. From the original sample of 12,795 drinking occasions, less than 5% of the students failed to answer some of the questions. Missing values were replaced by means of Markov Chain Monte Carlo estimates. A multiple imputation technique (as implemented in SPSS for Windows (version 20, IBM Corp., New York, NY, USA), performed five imputed datasets) was used to deal with all missing observations on the assumption that missing observations depend only on the observed covariates included in the model (MAR = missing at random assumption).

### 2.6. Statistical Analysis

The inter-correlations between the situational variables and motives were relatively low (r ≤ 0.22, *p* < 0.01), (Pearson correlation was used and the significance level was tested using a two-tailed test).

A mixed-effects linear regression model was constructed to address our research questions, because we are working with a two-level design with drinking occasions and their particular aspects at the first level, and students and their individual characteristics at the second level: i.e., 12,795 drinking occasions at level 1 nested within 4265 individuals at level 2. The primary outcome variable is the number of SDs per occasion. Because of a skewed response distribution, the number of SDs was log-transformed to approximate a normal distribution and reduce the impact of extreme values. As independent variables, we distinguished between the contextual variables (drinking day; time of the drinking day, duration of drinking, main purpose of drinking, drinking with food, drinking location, number of people who drink together, gender of people drinking together, relationship of the people drinking together) and the individual variables (gender, age, drinking motives). Studies have suggested that students from different disciplines may differ in their frequency and quantity of alcohol consumption [31]. Since the university/faculty was related to the outcome variables of interest in this sample, it was included as a covariate. The proportion of variance explained was computed using the method proposed by Snijders and Bosker [32]. Since there are large gender differences in drinking volume, the model was separately tested for men and women.

Since the time span over which the three drinking occasions were distributed varies between the students, another model including a variable indicating the time interval over which the three drinking occasions took place (e.g., 1 month/2 month/…) was tested. Due to the fact that a substantial number of students could not recollect at which time one or more of the three most recent drinking occasions took place, this analysis includes a far less large number of students (female = 417 students with 1251 drinking occasions; male = 1023 students with 3069 drinking occasions). The purpose of testing this model was to exploring whether the frequency of drinking occasions influences the effect of contextual variables and motives on drinking. Data of this model was not shown.

## 3. Results

Of the total number of 12,795 drinking occasions during the previous 12 months, 13.9% occurred within the previous week; 20.6% occurred between 2 weeks and 1 month ago, 18.8% occurred more than 1 month ago, and of the remaining 46.7% of the drinking occasions the students did not remember when they had happened. Comparison of the three most recent drinking occasions revealed that there was no significant difference in the standard drinking intake between the first, the second and the third most recent occasion. However, comparing occasions for which a date was reported with those the date was not reported, we found significant difference indicating a higher consumption in occasions with date was reported (Median of alcohol consumption per occasion with date reported was 3.4 (SD = 3.1) vs. with no date reported was 2.6 (SD = 2.7)). Table 1 shows that the participants scored higher on social motives, followed by conformity, and then by coping and enhancement motives.

Table 2 shows that the mean alcohol intake per occasion is 3.0 standard drinks (SD = 2.9) with a median of 2 standard drinks (interquartile range 1–4). Most of the drinking occasions take place in the weekends, in the evening/night, when having a party, drinks with food, and at home. Additionally, students drink in mostly mixed gender groups with 4–9 friends. Students rarely drink longer than 3 h per occasion.

Table 3 shows that, at the contextual level, both male and female students consumed more alcohol per occasion when the context is characterized by long duration of drinking occasion, large number of people and the participants being casual friends or acquaintances rather than family. Among male students only, more alcohol is drunk per occasion when they drink at a specific occasion such as a wedding or a celebration (as opposed to not a special event), when not having food, and when drinking in a tavern/pavement/outside (as opposed to a discotheque/karaoke bar/others). Additionally, male students drink less alcohol when they drink in exclusively female company. Among those variables, the duration of drinking has the strongest association. On average, the alcohol intake of students who spent ≥3 h drinking on an occasion was about two times higher than that of students who spent ≤1 h drinking on an occasion. 

At the individual level, all drinking motives were significantly associated with drinking among male students. Conformity motives were not significantly associated with drinking among women, while a higher score on conformity motives was associated with a significantly lower consumption among male students.

Individual characteristics explained 7% and 10% of the variance in alcohol intake per occasion among female and male students, respectively. Situational characteristics explained 11% and 23% of the variance in alcohol intake per occasion among female and male students, respectively. Together, the individual and contextual characteristics explained 16% and 27% of the variance in alcohol intake per occasion among female and male students, respectively. In a combined model, the contextual characteristics explained more variance (women 11% and men 23%) than individual characteristics (women 7%; men 10%) in alcohol intake per occasion. 

Controlling for the time span over which the three drinking occasions took place did not change anything in the effect of the contextual and individual variables on the outcome (number of glasses per occasion). Additionally, it confirmed our conclusion that drinking context variables explain more variance than individual ones (data not shown).

## 4. Discussion

Vietnamese students seem to consume less alcohol per drinking occasion than, for example, Canadian students (3.0 SDs vs. 4.4–4.5 SDs) [8,10] and probably than most students in European countries [1] or in the U.S. [33,34,35,36]. This finding is in line with another cross-countries study among students which showed that hazardous drinking is lower in Asia and Africa but higher in Australia, Europe, and North and South America [37].

The results of the present study indicate that, at the contextual level, drinking sessions lasting more than 3 h, drinking in large groups, and drinking with casual acquaintances rather than with family members, are associated with a significantly higher level of alcohol consumption. Drinking without food and drinking in a tavern/pavement/an outside location are likely to increase alcohol consumption per occasion among male students only. Findings at the contextual level are similar to those from studies among students in other countries such as in the U.S., Canada and the Netherlands, showing that students drink less when in the presence of a family member [38], that students are protected against alcohol problems when drinking with food [39], and that students drink more on occasions involving a larger group [9,10,13,16].

At the individual level, coping, social and enhancement motives are associated with a higher level of alcohol consumption among both genders, while conformity motives are associated with less alcohol consumption among male students. In this study, coping motives were most strongly positively associated with alcohol consumption of male and female students. Studies among adolescents in Switzerland, Canada, and the U.S. [40] similarly reported that enhancement and coping motives were positively related to alcohol use and to risky drinking in particular, and coping motives were additionally related to alcohol-related problems. In the present study, results at the individual level are also comparable with other studies among students/adolescents in European countries that show that coping motives are most strongly related to alcohol consumption and conformity motives are negatively related to alcohol consumption [27,28,41,42,43].

Both individual and contextual characteristics are important in explaining alcohol consumption per occasion. However, the variance explained by situational level variables is higher than that explained by individual level factors. A study among U.S. college students examined the relationship between individual and environmental variables and heavy alcohol use at the most recent drinking occasion; that study showed that environmental level characteristics explained more variance than the individual level predictors [13]. On the other hand, studies among Canadian students, in which the individual level explains 30.7% and situational level explains 25.2% of the variance, concluded that contextual level variables appear to be as important as individual level characteristics for the explanation of alcohol consumption per occasion [10]. However, it is difficult to compare our results with others because no study included drinking motives as an individual variable.

Our finding that contextual variables explain more of the variance might be an underestimation of the importance of motives. The correlation between contextual variables and motives was rather low (r < 0.22, *p* < 0.01). The low inter-correlations indicate little influence of motives on the selection of drinking situations. Beside that, as Cooper et al. suggest [44], we assume that the low correlation is mainly due to the measure of motives being “general”, whereas only a small number of specific drinking occasions was used to measure the contextual variables. One would expect that the response to a question like “How often do you drink to...” has a background related to a far larger number of drinking situations than the three most recent drinking occasions that we used to measure the context of the drinking situations.

Among students in Vietnam, contextual aspects explain more variance than individual characteristics in explaining alcohol consumption per occasion. In other words, the duration of a drinking session explains more of the actual consumption than the scores on social or enhancement motives. An explanation for this might be found in the Vietnamese drinking culture. Social motives and conformity motives score highest among Vietnamese students, indicating that motives aimed at adjusting to the situation, rather than motives maximizing individual benefits (enhancement; coping). This is different from most previously reported findings on motives from 13 other European countries [26,45], U.S. and Canada [35,40], where (besides social motives), enhancement motives score highest among adolescents and young persons.

In Vietnam, with the development of the economy, alcohol use has become a normal way to develop contacts, stimulate social interaction, and business discussions. In many regions in Vietnam, a frequently reported reason for drinking among Vietnamese people is peer pressure. People can only start to negotiate or work after drinking at least three “cups” of alcohol [18]. Of course, this drinking culture can also influence how young people drink. Vietnamese adolescents most often start to drink because of peer pressure, or based on imitation [46]. As a result, in contrast to other countries, in Vietnam conformity is far more important and enhancement is a less dominant drinking motive.

These findings have implications for alcohol prevention policies in Vietnam. Importantly, prevention should focus on detecting and discouraging Vietnamese students that score high on coping motives to help them reduce their alcohol consumption level. However, considering the relatively large influence of contextual variables on drinking, the main focus should be on prevention measures discouraging drinking for a long period per occasion, in taverns, at special events, in large groups, or with casual acquaintances. This implies an alcohol policy with general measures aiming to reduce both the frequency of certain drinking context and of alcohol consumption per occasion. Policies should aim to reduce the drinking duration per occasion by restricting the daily opening hours of alcohol outlets [47,48]. Additionally, shops/outlets selling alcohol should be closed during special events and have specific days of closure during public holidays. Moreover, a prevention policy should reduce the accessibility of alcohol in taverns/outside, by making a license a prerequisite for selling alcohol, even in a tavern [2,49]. Studies worldwide have shown that alcohol policies can effectively limit the availability of alcohol by restricting the opening hours of alcohol outlets/bars [50]; with government controls on the sale of alcohol [51], as well as increasing price [52] through increasing the tax on alcohol [53]. At the moment, in Vietnam a license is required only for selling spirits, but not for beer and wine [54]. Also, there is no policy related to restrictions for on/off premise sales of alcohol beverages related to hours, days, density, specific events/intoxicated persons, etc. [55]. Furthermore, there is no policy regarding the control of producing and selling home-brewed alcohol; this is a common type of alcohol in Vietnam [56] resulting in wide availability and accessibility throughout the country. The uncontrolled quality of alcohol also contributes to the low price of alcohol, increasing its availability and affordability.

## 5. Study Limitations

The present study required students to recall the three most recent drinking occasions, which is likely to lead to recall bias. The finding that, for those drinking occasions for which students could not specify how long ago they had occurred, the consumption was significantly lower than for those occasions that students could (still) remember how long ago they occurred, is an indication that there is some recall bias. Additionally, the study tested the model with only 2 individual variables (drinking motives and age) and 10 situational variables. The situational variables focused upon aspects of the direct drinking context. A limitation was not only unable to include other aspects of drinking context which may possibly influence drinking such as loudness of music and crowdedness [15,16,17] but also did not include other environmental aspects such as aspects of neighborhoods in which the drinking took placed (e.g., urban vs. sub-urban or rural). It is possible that the results may be different if other individual characteristics possibly determining consumption, had been included, e.g., expectancies, school results. Yet, the fact that motives have been shown to mediate other individual characteristics [22,23,24,25] may mean that, even when including other individual characteristics, the balance between the amount of variance explained by contextual respectively, individual characteristics might not change a lot. However, it should be mentioned that drinking motives vary from day to day as a function of mood [57]. Therefore, examining the association of daily drinking motives at the drinking occasion level may explain an additional part of variance in alcohol intake. 

Furthermore, in our study, motives and context were measured on a different level. When motives are measured within a situation, this might change the amount of variance explained by both context and motives. Longitudinal studies with many more than three drinking occasions are required to further elucidate the interaction between motives and contextual variables. In addition, the generalizability of the results is limited since the study included Vietnam only.

## 6. Conclusions

The results of this study contribute to the empirical evidence on alcohol intake among students, suggesting that drinking context more strongly predict alcohol consumption than drinking motives. Prevention programs should incorporate these findings within a Vietnamese cultural context by looking at both the individual level variables (people in whom coping motives are dominant) and contextual level factors (e.g., restricting alcohol availability, and its accessibility and affordability should receive attention).

## Figures and Tables

**Table 1:** Demographic and individual characteristics of drinkers (*n* = 4265).

Individual Variables		*n* = 4265	%
University			
	Medical	1422	33.3
	Technology	1522	35.7
	Economics	1321	31.0
Province			
	Hanoi	1202	28.2
	BMT	1073	25.2
	HCM	1021	23.9
	Hue	969	22.7
Gender			
	Male	2556	59.9
	Female	1675	39.3
	Missing	34	0.8
Drinking motives		Median	(IQR)
	Average score of Social	3.2	(2.4, 3.8)
	Average score of Conformity	1.8	(1.4, 2.4)
	Average score of Coping	1.4	(1.0, 1.8)
	Average score of Enhancement	1.4	(1.0, 2.0)
	Age (years)	Mean = 20.7	SD = 1.7

**Table 2:** Situational characteristics of 12,795 drinking occasions among 12 universities/faculties in four provinces in Vietnam.

Situational Variables		*n* (%)	Situational Variables		*n* (%)
Drinking day			Location of drinking		
	Working day	3591 (28.1)		Home	6328 (50.0)
	Weekend	5075 (39.7)		Restaurant	3522 (27.8)
	Holidays	3996 (31.2)		Tavern/pavement/outside	2274 (18.0)
	Missing value	133 (1.0)		Discotheque/karaoke bar/others	536 (4.2)
Time of drinking day				Missing value	135 (1.1)
	Morning/lunch	2119 (16.7)	Number of group members		
	Afternoon	1605 (12.7)		0	170 (1.3)
	Evening/midnight	8962 (70.6)		1–3	3089 (24.1)
	Missing value	109 (0.9)		4–9	6011 (47.0)
Drinking duration				more than 10	3389 (26.5)
	<1 h	3658 (28.9)		Missing value	136 (1.1)
	1–2 h	4472 (35.3)	Gender of group members		
	2–3 h	3294 (26.0)		Female only	640 (5.0)
	More than 3 h	1241 (9.7)		Male only	3742 (29.2)
	Missing value	130 (1.0)		Mixed gender	8117 (63.4)
Main purpose of drinking				Missing value	296 (2.3)
	Party	5634 (44.6)	Relationship of group member		
	Meeting with friends	4508 (35.7)		Family/relative relationship	2265 (18.1)
	Wedding/celebration	1452 (11.5)		Friend relationship	8959 (70.0)
	Special event	1041 (8.1)		Casual relationship	1262 (10.1)
	Missing value	160 (1.3)		Missing value	309 (2.4)
Drinking with food (even snack)			Outcome variables		
	Yes	12298 (96.1)		Standard drinks per occasion	Mean (SD) = 3.0 (2.9)
	No	254 (2.0)		Standard drinks per occasion	Median (IQR) = 2 (1, 4)
	Missing value	243 (1.9)		Missing value	619 (4.8)

**Table 3:** Multilevel estimates for alcohol intake per occasion.

	Variables		Standard Beta Coefficient	
			Male Students	Female Students
			Constant = 0.66	Constant = −0.28
Control variables	University (reference, Economics)			
		Medical	−0.09 **	−0.13 **
		Technology	0.06	0.00
	Province (reference, Hue)			
		Hanoi	−0.12 ***	−0.1 *
		BMT	0.03	0.06
		HCM	−0.10 **	0.06
Situational variables	Drinking day (reference, vacations)			
		Week day	0.03	−0.03
		Weekend	0.02	0.08
	Time of drinking day (reference, evening/midnight)			
		Morning/lunch	−0.03	−0.02
		Afternoon	−0.00	−0.02
	Drinking duration (reference over 3 h)			
		<1 h	−0.81 ***	−0.59 ***
		1–2 h	−0.46 ***	−0.38 ***
		2–3 h	−0.21 ***	−0.19 ***
	Main purpose of drinking (reference, wedding or celebration)			
		Party	−0.02	0.00
		Meeting with friends	−0.03	0.02
		Not special event	−0.17 ***	−0.08
	Drinking with food (even snack) (reference, have food)			
		No	0.2 ***	0.06
	Location (reference, discotheque/karaoke bar/others)			
		Home	0.03	0.05
		Restaurant	0.06	0.04
		Tavern/pavement/outside	0.01 **	0.07
	Number of group members (reference, more than 10 people)			
		0	0.03	−0.01
		1–3	−0.13 ***	−0.04
		4–9	−0.06 ***	−0.04 *
	Drinking company (reference, mixed gender)			
		Female only	−0.16 **	0.04
		Male only	0.02	−0.01
	Relationship (reference, casual relationship)			
		Family member/relative	−0.17 ***	−0.13 **
		Friend relationship	0.04	−0.03
	Occasion (reference, third most recent occasion)			
		First recent occasion	−0.01	−0.01
		Second recent occasion	−0.02	−0.00
Individual variables	Age		0.016 *	0.02
	Drinking motives			
		Social	0.10 ***	0.09 ***
		Enhancement	0.05 **	0.08 **
		Coping	0.13 ***	0.13 ***
		Conformity	−0.04 *	0.00
Statistics	R square explained by individual variables		0.10	0.07
	R square explained by situational variables		0.23	0.11
	R square explained by both individual and situational variables		0.27	0.16

* *p* < 0.05; ** *p* < 0.01; *** *p* < 0.001.
